# Description of two species of the orb-weaver spider genus *Argiope* Audouin, 1826 (Araneae, Araneidae) from Xizang, China

**DOI:** 10.3897/BDJ.12.e125601

**Published:** 2024-07-04

**Authors:** Mi Xiaoqi, Tong Zhang, Cheng Wang

**Affiliations:** 1 College of Agriculture and Forestry Engineering and Planning, Guizhou Provincial Key Laboratory for Biodiversity Conservation and Utilization in the Fanjing Mountain Region, Tongren University, Tongren, China College of Agriculture and Forestry Engineering and Planning, Guizhou Provincial Key Laboratory for Biodiversity Conservation and Utilization in the Fanjing Mountain Region, Tongren University Tongren China; 2 Central South Inventory and Planning Institute of National Forestry and Grassland Administration, Changsha, China Central South Inventory and Planning Institute of National Forestry and Grassland Administration Changsha China

**Keywords:** Taxonomy, Argiopinae, sexual dimorphism, DNA barcoding

## Abstract

**Background:**

The spider genus *Argiope* Audouin, 1826, comprises 88 species worldwide, including 23 species occurring in China. Two *Argiope* species were collected by the spider survey on Yarlung Zangbo Grand Canyon National Nature Reserve, Xizang, southwest China, conducted in 2023.

**New information:**

Two species of the orb-weaver spider genus *Argiope* from Xizang, China are described, including a new species, *A.beibeng* Mi & Wang, **sp. nov.** (♂♀) and a known species, *A.caesarea* Thorell, 1897 (♂♀). The unknown male of *A.caesarea* is described for the first time.

## Introduction

The orb-weaver spider genus *Argiope* is characterised by their showy colorful females and their unique web stabilimenta (Levi 1983, Tan 2018). As one of the most typical extremely sexual dimorphic genera, it is hard to match the females to males, for example, among the 88 known *Argiope* species worldwide, 28 are only known from females, and three are only known from males (World Spider Catalog 2024).

Levi (1983) revised the *Argiope* species from the western Pacific region, a total of 49 species were reported and divided into seven species groups, including eight species from China. Yin et al. (1997) provided a comprehensive study of the Chinese *Argiope* species, described and illustrated 19 *Argiope* species and divided 18 into Levi’s seven species groups, leaving *A.lobata* not assigned to any group (Table 1). Wang et al. (2021) taxonomic revised three *Argiope* species based on morphological and/or molecular evidence and added two newly recorded species from China.

While examining the *Argiope* specimens from Yarlung Zangbo Grand Canyon National Nature Reserve, Xizang, southwest China, *Argiopecaesarea* Thorell, 1897, and a new species, were identified. The goals of the present paper are to describe the new species, and the unknown male of *A.caesarea*, and to map those species.

## Materials and methods

All the specimens were collected by beating shrubs or hand collecting and are preserved in 75% ethanol. All the specimens are deposited in the Museum of Tongren University, China (TRU). The specimens were examined with an Olympus SZX16 stereomicroscope. The epigynes were cleared in lactic acid for examination and imaging. The left male pedipalp was dissected in ethanol for examination, description, and imaging. Photographs of the habitus and copulatory organs were taken with a Kuy Nice digital camera mounted on an Olympus BX43 com pound microscope. Compound focus images were generated using Helicon Focus v. 6.7.1. All measurements are given in millimeters. Leg measurements are given as total length (femur, patella + tibia, metatarsus, tarsus). References to figures in the cited papers are listed in lowercase type (fig. or figs), and figures in this paper are noted with an initial capital (Fig. or Figs).

A partial fragment of the mitochondrial cytochrome oxidase subunit I (COI) gene of the two species was amplified and sequenced using the primers LCOI1490 and HCOI2198 (Folmer et al. 1994). The accession numbers are provided (Table 2). The pairwise genetic distances (Kimura two-parameter [K2P]) (Table 3) were calculated using MEGA 6.0 to assess the genetic differences.

Abbreviations used in the text and figures are as follows: **C** conductor; **CD** copulatory duct; **CO** copulatory opening; **Cr** crest; **E** embolus; **FD** fertilization duct; **MA** median apophysis; **Pc** paracymbium; **PP** posterior plate; **R** rim; **S** septum; **Sp** spermatheca.

## Taxon treatments

### Argiope beibeng

Mi & Wang, sp. nov.

4B0E4C0C-C15E-5ED0-8460-F7DA2478EC10

0B899278-34C3-491C-B967-B19C2C8C38F6

#### Materials

- **Type status:** Holotype. **Occurrence:** recordedBy: Xiaoqi Mi et al.; individualID: TRU-Araneidae-315; sex: male; associatedSequences: PP810205; occurrenceID: DC00441F-8EF7-5A10-AB5A-3171BB006A78; **Location:** country: China; stateProvince: Xizang Autonomous Region; county: Medog; locality: Beibeng Township, Jiangxin Village; verbatimElevation: ca 700 m; verbatimLatitude: 29°13.70′N; verbatimLongitude: 95°8.04′E; **Identification:** identifiedBy: Xiaoqi Mi; **Event:** samplingProtocol: hand collecting; year: 2023; month: 8; day: 17
- **Type status:** Paratype. **Occurrence:** recordedBy: Xiaoqi Mi et al.; individualID: TRU-Araneidae-316; individualCount: 1; sex: female; associatedSequences: PP899059; occurrenceID: 7899DD6A-FBB9-5032-A69B-54FBB23F7FE7; **Location:** country: China; stateProvince: Xizang Autonomous Region; county: Medog; locality: Beibeng Township, Jiangxin Village; verbatimElevation: ca 700 m; verbatimLatitude: 29°13.70′N; verbatimLongitude: 95°8.04′E; **Identification:** identifiedBy: Xiaoqi Mi; **Event:** samplingProtocol: hand collecting; year: 2023; month: 8; day: 17

#### Description

**Figure 1.**
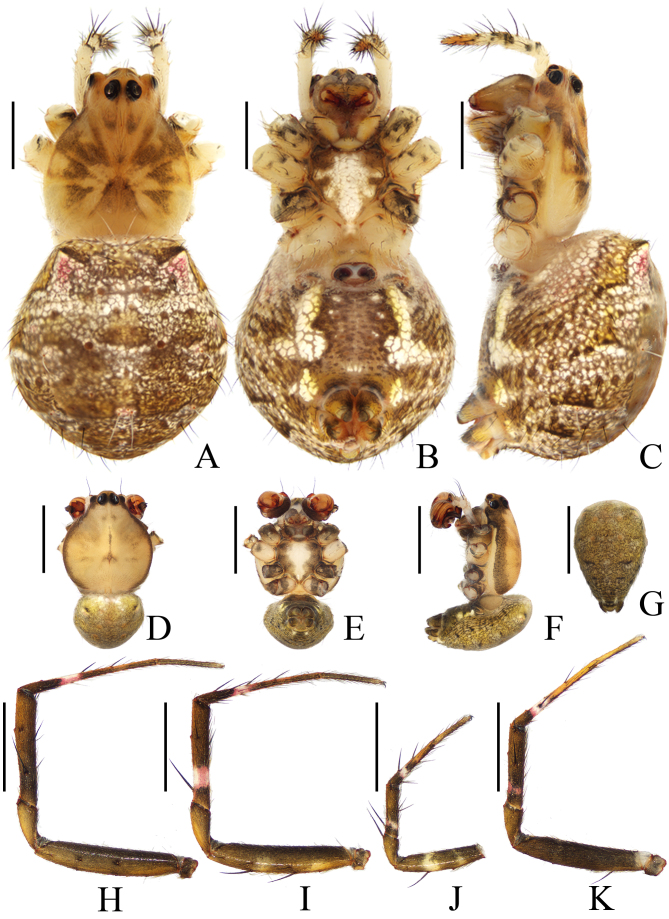
*Argiopebeibeng* Mi & Wang, sp. nov. **A**–**C** female paratype TRU-Araneidae-316, **D**–**K** male holotype TRU-Araneidae-315. **A**, **D** habitus, dorsal view; **B**, **E** ibid., ventral view; **C**, **F** ibid., lateral view; **G** abdomen, posterior view **H** leg I, prolateral view; **I** leg II, prolateral view; **J** leg III, prolateral view; **K** leg IV, prolateral view. Scale bars: 1.0 mm.

**Male** (TRU-Araneidae-315). Total length 2.35. Carapace 1.50 long, 1.35 wide; abdomen 1.60 long, 1.05 wide. Eye sizes and interdistances: AME 0.11, ALE 0.05, PME 0.10, PLE 0.08, AME–AME 0.05, AME–ALE 0.03, PME–PME 0.15, PME–PLE 0.15. Legs: I 5.70 (1.60, 1.80, 1.55, 0.75), II 5.40 (1.55, 1.70, 1.45, 0.70), III 2.95 (0.95, 0.90, 0.65, 0.45), IV 4.40 (1.45, 1.30, 1.05, 0.60). Carapace (Fig. 1D, F) acutely narrowed in cephalic region and rounded in thorax region, pale with a longitudinal brown stripe medially and brown margins. Fovea depressed. Chelicerae (Fig. 1E, F) yellowish brown, with four promarginal and three retromarginal teeth. Endites (Fig. 1E) dark brown at base and pale on the inner side. Labium (Fig. 1E) dark brown at base and pale at tip. Sternum (Fig. 1E) heart-shaped, pale medially and dark brown laterally. Legs (Fig. 1H–K) dark brown to greenish brown, with pale annuli, armed with macrosetae. Abdomen (Fig. 1D–G) oval, yellowish green with four pairs of brown patches. Venter abdomen dark brown, with a pair of white patches median-laterally. Spinnerets dark brown.

**Figure 2.**
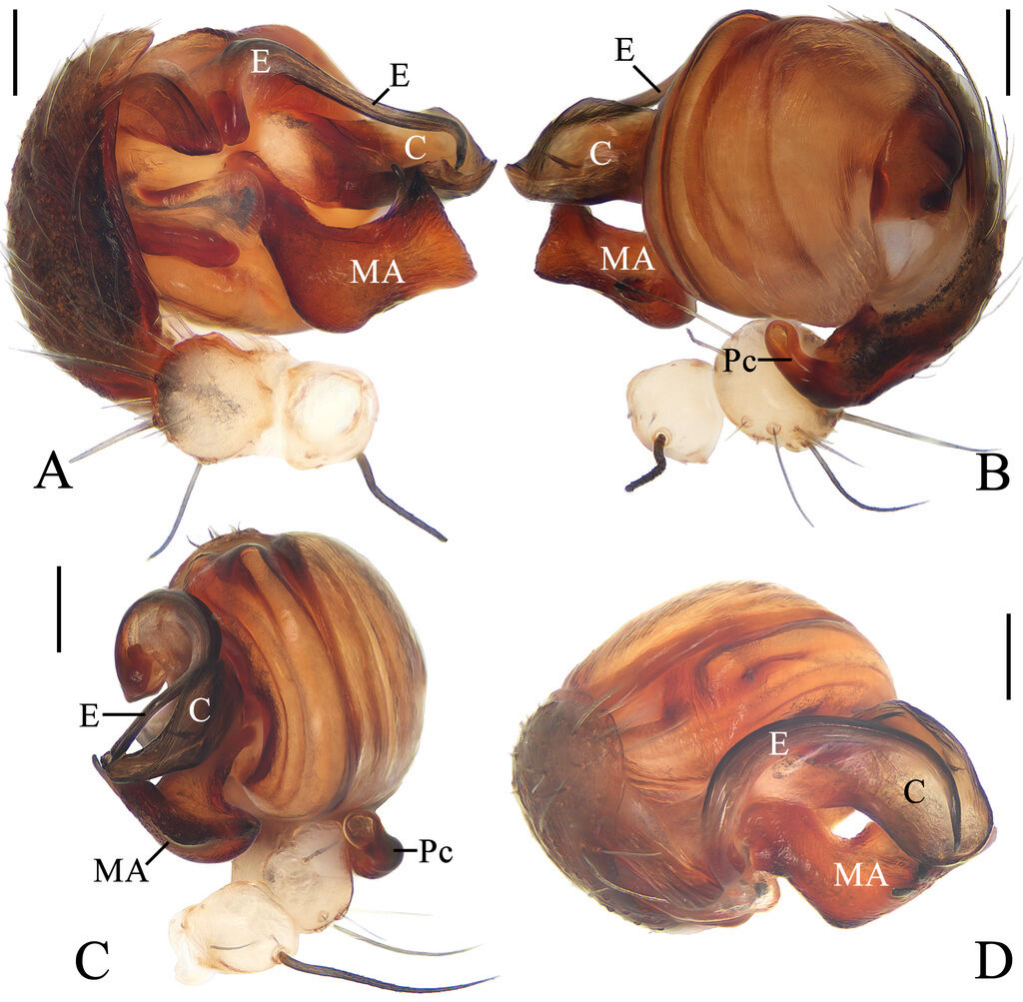
*Argiopebeibeng* Mi & Wang, sp. nov. male holotype TRU-Araneidae-315. **A** pedipalp, prolateral view **B** ibid., retrolateral view **C** ibid., ventral view **D** ibid., apical view. Scale bars: 0.1 mm.

**Pedipalp** (Fig. 2A–D): patella with a long bristle; tibia swollen; paracymbium fingerlike; median apophysis boot shaped, with a fine dorsal spur near the distal end; conductor about equal length to median apophysis, broad and slightly curled; embolus tapered, curved to C-shaped in apical view.

**Female** (TRU-Araneidae-316). Total length 5.80. Carapace 2.70 long, 2.20 wide; abdomen 3.40 long, 3.00 wide. Eye sizes and interdistances: AME 0.15, ALE 0.08, PME 0.13, PLE 0.10, AME–AME 0.13, AME–ALE 0.13, PME–PME 0.28, PME–PLE 0.30. Legs: I 10.55 (3.10, 3.45, 2.95, 1.05), II 9.80 (3.30, 3.15, 2.35, 1.00), III 6.15 (2.05, 1.85, 1.45, 0.80), IV 9.65 (3.25, 3.00, 2.50, 0.90). Carapace (Fig. 1A) pear-shaped, pale yellow with brown radial markings. Fovea depressed. Chelicerae (Fig. 1B, C) grayish yellow with four promarginal and three retromarginal teeth. Endites and labium (Fig. 1B) dark at base and pale apically. Sternum (Fig. 1B) heart-shaped, yellow with a wide longitudinal branched white band. Legs grayish brown with yellow annuli. Abdomen (Fig. 1A–C) pentagonal, with a pair of anterio-lateral low humps, dorsum grayish brown with silver to pink spots. Venter abdomen grayish brown, with two pairs of white patches laterally and three pairs of white spots medially. Spinnerets grayish yellow.

**Figure 3.**
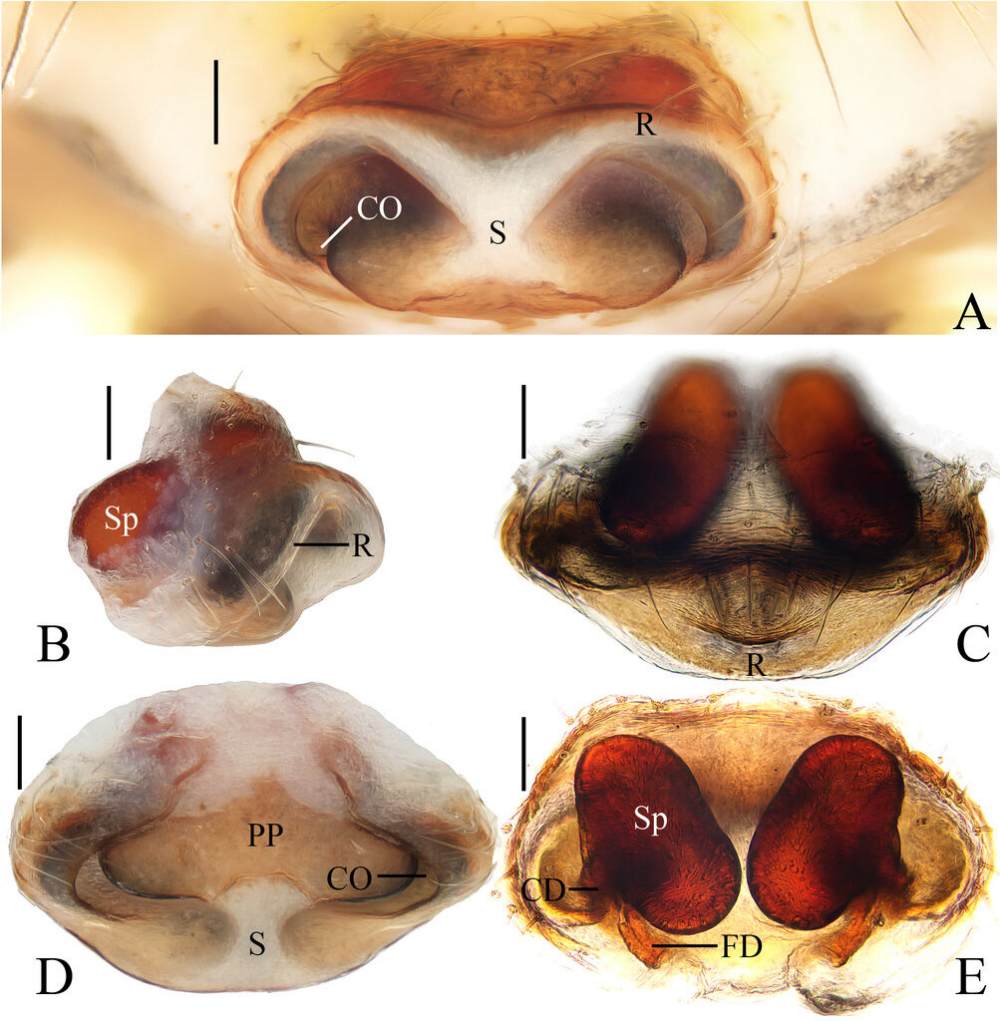
*Argiopebeibeng* Mi & Wang, sp. nov. female paratype TRU-Araneidae-316. **A** epigyne, ventral view **B** ibid., lateral view **C** ibid., anterior view **D** ibid., posterior view **E** vulva, dorsal view. Scale bars: 0.1 mm.

**Epigyne** (Fig. 3A–E) rimmed anteriorly and laterally; median septum narrowest at the middle part; posterior plate wide, about 2/3 the epigynal width; copulatory openings located on each side of the posterior plate in posterior view; copulatory ducts shorter than half the spermathecal length; spermathecae reniform, posterior end nearly touch, anterior end directed downward.

#### Diagnosis

The male of this species resembles *A.anasuja* Thorell, 1887 in having similar embolus, but differs in: 1) distal end of the median apophysis about equal width to pedipalp tibia (Fig. 2A), versus about half the width of pedipalp tibia in *A.anasuja* (Levi 1983: fig. 172); and 2) distal end of the median apophysis partly covers the conductor in prolatertal view (Fig. 2A), versus far away from the conductor in *A.anasuja* (Levi 1983: fig. 172). The female of this species resembles *A.hoiseni* Tan, 2018 in appearance, but differs in: 1) posterior plate about 2/3 width of the epigynum (Fig. 3D), versus about 1/2 in *A.hoiseni* (Tan 2018: fig. 11); and 2) anterior end of spermathecae directed downward (Fig. 3B), versus directed upward in *A.hoiseni* (Tan 2018: fig. 10).

#### Etymology

The specific name is a noun in apposition and refers to the type locality.

#### Distribution

China (Xizang) (Fig. 7).

#### Notes

Matching of male and female was justified by DNA barcoding.

### Argiope caesarea

Thorell, 1897

60FB2330-DFAB-50C3-B90D-3CD177F18966

#### Materials

- **Type status:** Other material. **Occurrence:** recordedBy: Xiaoqi Mi et al.; individualID: TRU-Araneidae-317–318; sex: 1 male, 1 female; occurrenceID: 6FA32B3E-0A35-54E0-A122-D608290F770C; **Location:** country: China; stateProvince: Xizang Autonomous Region; county: Medog; locality: Beibeng Township, De’ergong Village; verbatimElevation: ca 1510 m; verbatimLatitude: 29°12.46′N; verbatimLongitude: 95°9.39′E; **Identification:** identifiedBy: Xiaoqi Mi; identificationReferences: Levi 1983; Yin et al. 1997; **Event:** samplingProtocol: hand collceting; year: 2023; month: 8; day: 14
- **Type status:** Other material. **Occurrence:** recordedBy: Xiaoqi Mi et al.; individualID: TRU-Araneidae-319; individualCount: 1; sex: male; occurrenceID: 823D7CF3-3A37-5996-AA57-381E97CEC1B4; **Location:** country: China; stateProvince: Xizang Autonomous Region; county: Medog; locality: Beibeng Township, De’ergong Village; verbatimElevation: ca 1700 m; verbatimLatitude: 29°10.81′N; verbatimLongitude: 95°8.51′E; **Identification:** identifiedBy: Xiaoqi Mi; identificationReferences: Levi 1983; Yin et al. 1997; **Event:** samplingProtocol: beating shrubs; year: 2023; month: 8; day: 15
- **Type status:** Other material. **Occurrence:** recordedBy: Xiaoqi Mi et al.; individualID: TRU-Araneidae-320–321; individualCount: 2; sex: females; occurrenceID: D9B009DB-2D17-5DB0-9F72-8E3759781A13; **Location:** country: China; stateProvince: Xizang Autonomous Region; county: Medog; locality: Beibeng Townsip, Xigong resettlement areas; verbatimElevation: ca 1130 m; verbatimLatitude: 29°14.57′N; verbatimLongitude: 95°14.82′E; **Identification:** identifiedBy: Xiaoqi Mi; identificationReferences: Levi 1983; Yin et al. 1997; **Event:** samplingProtocol: hand collceting; year: 2023; month: 8; day: 15
- **Type status:** Other material. **Occurrence:** recordedBy: Xiaoqi Mi et al.; individualID: TRU-Araneidae-322; individualCount: 1; sex: male; occurrenceID: AD3090C1-C4B3-543D-95D6-18625B591C02; **Location:** country: China; stateProvince: Xizang Autonomous Region; county: Medog; locality: Beibeng Townsip, Xigong resettlement areas; verbatimElevation: ca 1560 m; verbatimLatitude: 29°12.36′N; verbatimLongitude: 95°19.07′E; **Identification:** identifiedBy: Xiaoqi Mi; identificationReferences: Levi 1983; Yin et al. 1997; **Event:** samplingProtocol: beating shrubs; year: 2023; month: 8; day: 17
- **Type status:** Other material. **Occurrence:** recordedBy: Xiaoqi Mi et al.; individualID: TRU-Araneidae-323; individualCount: 1; sex: male; occurrenceID: 00532F2D-CAB1-5E75-A237-6E165F0C6B6A; **Location:** country: China; stateProvince: Xizang Autonomous Region; county: Medog; locality: Dexing Township, Deguo Village; verbatimElevation: ca 780 m; verbatimLatitude: 29°23.81′N; verbatimLongitude: 95°22.35′E; **Identification:** identifiedBy: Xiaoqi Mi; identificationReferences: Levi 1983; Yin et al. 1997; **Event:** samplingProtocol: beating shrubs; year: 2023; month: 8; day: 20
- **Type status:** Other material. **Occurrence:** recordedBy: Cheng Wang, Hong Yao; individualID: TRU-Araneidae-324–325; individualCount: 2; sex: males; occurrenceID: 2E2D8B75-306B-5E26-87EC-8DDA08CF2018; **Location:** country: China; stateProvince: Xizang Autonomous Region; county: Medog; locality: Damu Township, along the G219 road from Kabu Village to Damu Village; verbatimElevation: ca 1380 m; verbatimLatitude: 29°28.70′N; verbatimLongitude: 95°27.20′E; **Identification:** identifiedBy: Xiaoqi Mi; identificationReferences: Levi 1983; Yin et al. 1997; **Event:** samplingProtocol: beating shrubs; year: 2023; month: 8; day: 19

#### Description

**Figure 4.**
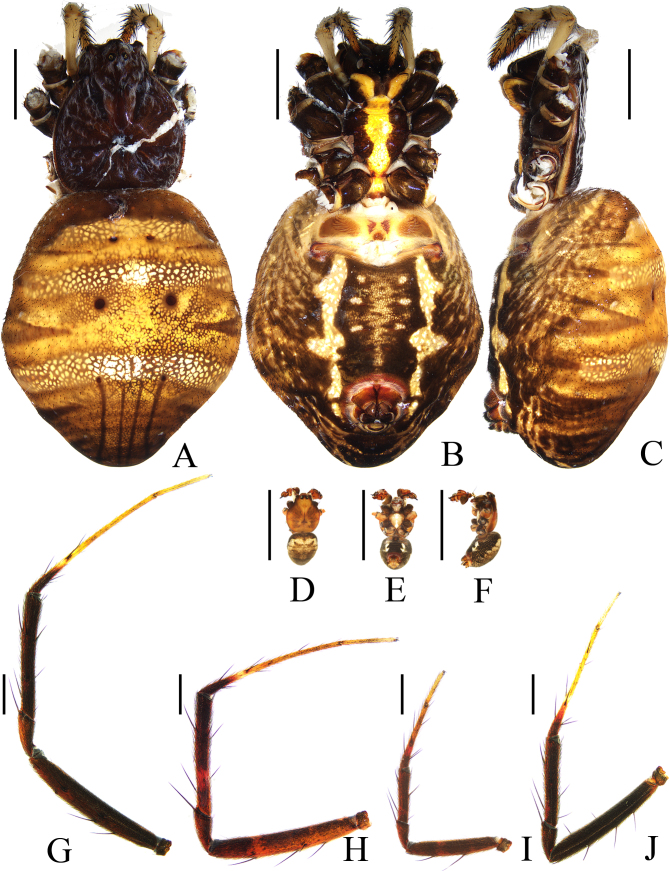
*Argiopecaesarea* Thorell, 1897 **A**–**C** female TRU-Araneidae-318, **D**–**K** male TRU-Araneidae-317. **A**, **D** habitus, dorsal view; **B**, **E** ibid., ventral view; **C**, **F** ibid., lateral view; **G** leg I, prolateral view; **H** leg II, prolateral view; **I** leg III, prolateral view; **J** leg IV, prolateral view. Scale bars: 5.0 mm (A–F); 1.0 mm (G–J).

**Male** (TRU-Araneidae-317). Total length 5.10. Carapace 2.80 long, 2.30 wide; abdomen 3.10 long, 2.10 wide. Eye sizes and interdistances: AME 0.15, ALE 0.10, PME 0.15, PLE 0.13, AME–AME 0.15, AME–ALE 0.10, PME–PME 0.25, PME–PLE 0.20. Legs: I 13.20 (3.80, 4.00, 3.90, 1.50), II 12.80 (3.80, 4.00, 3.70, 1.30), III 6.80 (2.20, 2.00, 1.70, 0.90), IV 10.60 (3.50, 3.10, 3.00, 1.00). Carapace (Fig. 4D) acutely narrowed in cephalic region and rounded in thorax region, yellowish brown with dark brown radial markings. Fovea depressed. Chelicerae (Fig. 4E, F) yellowish brown, with four promarginal and two retromarginal teeth. Endites and labium (Fig. 4E) dark brown at base, pale at tip. Sternum (Fig. 4E) heart-shaped, yellow with white patches medially. Legs (Fig. 4G–J) yellow to dark brown, spiny. Abdomen (Fig. 4D–F) oval, dorsum grayish brown with pale patches. Venter abdomen dark with a pair of white patches laterally. Spinnerets reddish brown.

**Figure 5.**
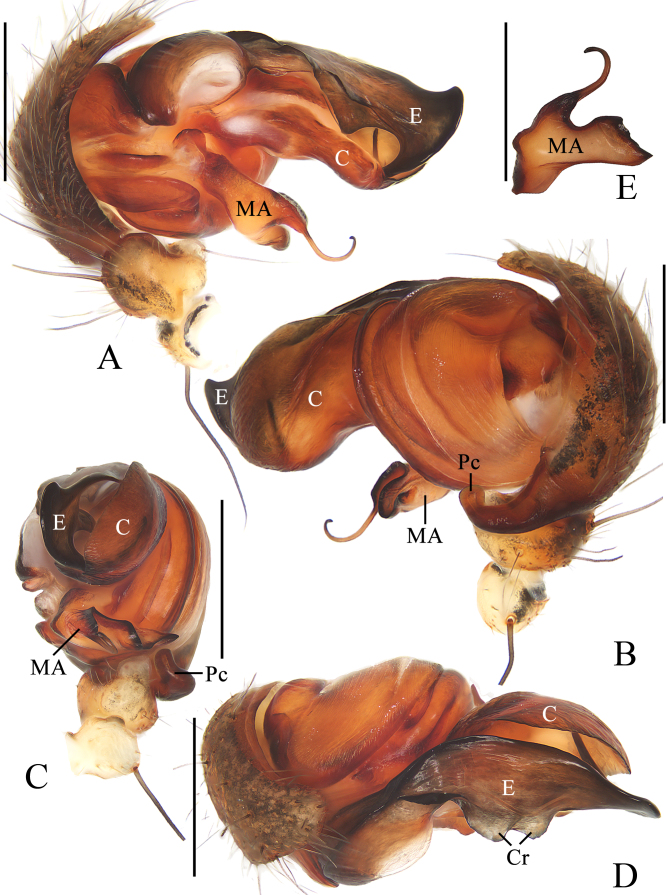
*Argiopecaesarea* Thorell, 1897 male TRU-Araneidae-317. **A** pedipalp, prolateral view **B** ibid., retrolateral view **C** ibid., ventral view **D** ibid., apical view **E** median apophysis. Scale bars: 1.0 mm.

**Pedipalp** (Fig. 5A–E) patella with a long bristle; tibia swollen; paracymbium fingerlike; median apophysis bifurcated, dorsal ramus filamentous distally; conductor flat, slightly curled; embolus extended at middle part, ventral surface with two crests, distal end coiled.

**Female** (TRU-Araneidae-318). Total length 30.10. Carapace 11.10 long, 9.00 wide; abdomen 20.20 long, 17.40 wide. Eye sizes and interdistances: AME 0.40, ALE 0.25, PME 0.35, PLE 0.35, AME–AME 0.40, AME–ALE 0.90, PME–PME 0.75, PME–PLE 1.50. Legs: I 51.60 (16.30, 15.80, 16.20, 3.30), II 50.70 (15.80, 15.90, 16.00, 3.00), III 31.20 (11.20, 9.40, 8.20, 2.40), IV 48.30 (17.20, 14.50, 14.10, 2.50). Carapace (Fig. 4A) narrowed in cephalic region and rounded in thorax region, dark brown, covered with pale hairs. Fovea depressed. Chelicerae (Fig. 4B, C) dark bown, with four promarginal and three retromarginal teeth. Endites (Fig. 4B) dark at base, pale on the inner side. Labium (Fig. 4B) pale with dark brown lateral edges. Sternum (Fig. 4B) heart-shaped, dark brown laterally, with a longitudinal white constricted band. Legs dark brown without annuli. Abdomen (Fig. 4A–C) pentagonal, truncated broadly in front and slightly pointed posteriorly, dorsal yellowish brown with three whitish transverse bands. Venter abdomen grayish-brown with a pair of white longitudinal bands laterally and three pairs of white spots in the middle. Spinnerets reddish brown.

**Figure 6.**
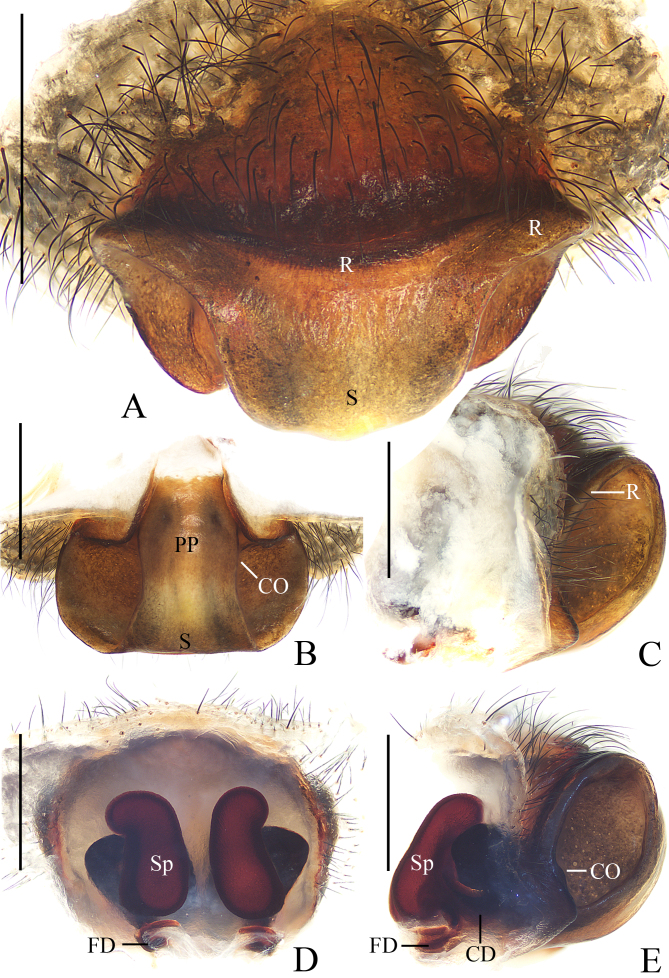
*Argiopecaesarea* Thorell, 1897 female TRU-Araneidae-318. **A** epigyne, ventral view **B** ibid., posterior view **C** ibid., lateral view **D** vulva, dorsal view **E** ibid., lateral view. Scale bars: 1.0 mm.

**Epigyne** (Fig. 6A–E) wider than long, rimmed anteriorly and laterally, with a wide septum; posterior plate with parallel edges, almost 1/3 the epigynal width; copulatory openings located on each side of the posterior plate in posterior view; copulatory ducts about equal length to the spermathecae; spermathecae reniform, separated, anterior end directed downward.

#### Diagnosis

The male of this species resembles *A.pulchella* Thorell, 1881 and *A.versicolor* (Doleschall, 1859) in general shape of the pedipalp, but differs in: 1) ventral surface of middle embolus with two crests (Fig. 5D) versus lacking in *A.pulchella* (Levi 1983: fig. 248) and *A.pulchella* (Levi 1983: fig. 259); 2) embolus lacking a pendant (Fig. 5A) versus with a pendant in *A.pulchella* (Levi 1983: fig. 248) and *A.pulchella* (Levi 1983: fig. 259); 3) tip of embolus coiled (Fig. 5A–D) versus curved in *A.pulchella* (Levi 1983: fig. 249) and *A.pulchella* (Levi 1983: fig. 260); and 4) median apophysis spur filamentous (Fig. 5A, B, E) versus thorn-shaped in *A.pulchella* (Levi 1983: fig. 249) and *A.pulchella* (Levi 1983: fig. 260). The female of this species closely resembles *A.amoena* L. Koch, 1878 in appearance, but differs in: 1) median septum wider in anterior than posterior (Fig. 6B) versus about equal width in *A.amoena* (Yin et al. 1997: fig. 6c); 2) median septum not constricted (Fig. 6A, B) versus constricted in middle part in *A.amoena* (Yin et al. 1997: fig. 6c); and 3) dorsal abdomen lacking white spots (Fig. 4A) versus middle transverse band with five white spots presented in *A.amoena* (Yin et al. 1997: fig. 6a).

#### Distribution

**Figure 7.**
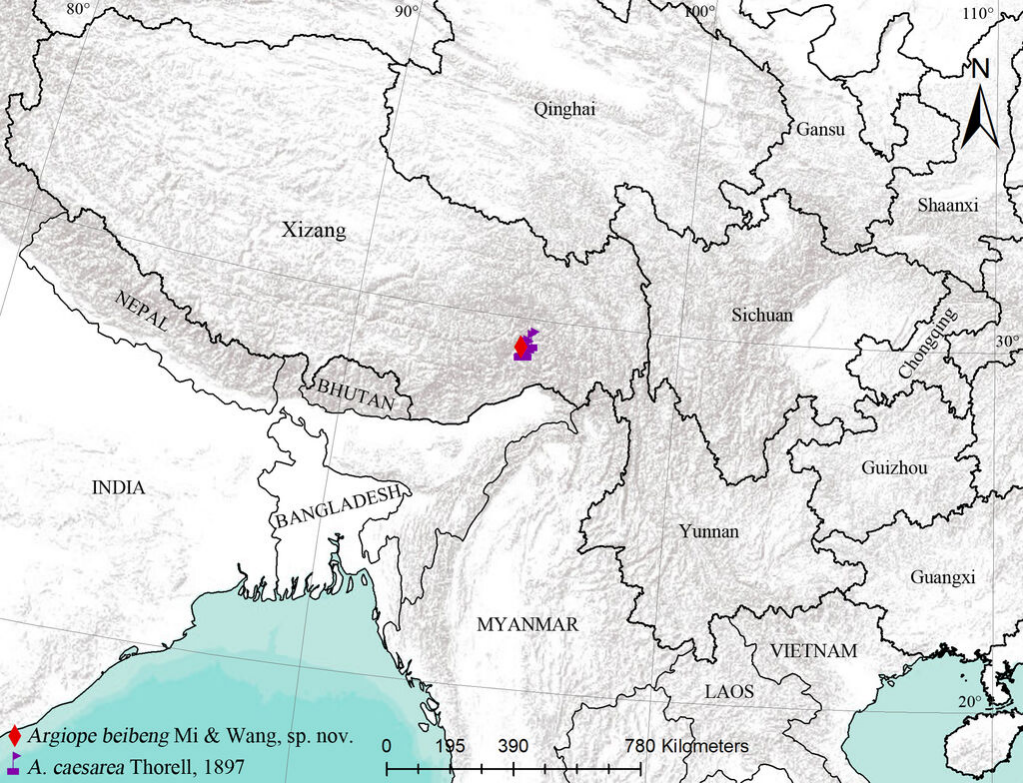
Distributional records of *Argiope* species.

China - Xizang, Yunnan (Fig. 7), India, Myanmar.

#### Notes

Female TRU-Araneidae-318 and male TRU-Araneidae-317 were collected in the same web, and matching of male and female was also justified by DNA barcoding.

## Supplementary Material

XML Treatment for Argiope beibeng

XML Treatment for Argiope caesarea

## Figures and Tables

**Table 1.** Yin et al. (1997) grouping on 18 *Argiope* species.

Species group	Included species
*A.ocula* group	*A.ocula* Fox, 1938 and *A.macrochoera* Thorell, 1891 (misidentification, female of *A.cameloides* Zhu & Song, 1994 according Wang et al. 2021)
*A.aemula* group	*A.aemula* (Walckenaer, 1841) and *A.catenulata* (Doleschall, 1859)
*A.caesarea* group	*A.caesarea* Thorell, 1897
*A.amoena* group	*A.amoena* L. Koch, 1878, *A.boesenbergi* Levi, 1983 and *A.bruennichi* (Scopoli, 1772)
*A.trifasciata* group	*A.trifasciata* (Forsskål, 1775)
*A.minuta* group	*A.minuta* Karsch, 1879 and *A.perforata* Schenkel, 1963
*A.aetherea* group	*A.aetherea* (Walckenaer, 1841), *A.aetheroides* Yin, Wang, Zhang, Peng & Chen, 1989, *A.pulchella* Thorell, 1881, *A.pulchelloides* Yin, Wang, Zhang, Peng & Chen, 1989, *A.versicolor* (Doleschall, 1859), *A.cameloides* and *A.jinghongensis* Yin, Peng & Wang, 1994

**Table 2.** Voucher specimen information.

Species	Voucher code	Sex	GenBank accession number
*Argiopebeibeng* Mi & Wang, sp. nov.	TRU-Araneidae-315	♂	PP810205
*A.beibeng* Mi & Wang, sp. nov.	TRU-Araneidae-316	♀	PP899059
*A.caesarea* Thorell, 1897	TRU-Araneidae-317	♂	PP808887
*A.caesarea* Thorell, 1897	TRU-Araneidae-318	♀	PP808888

**Table 3.** Intraspecific and interspecific nucleotide divergences for two *Argiope* species using Kimura two-parameter model.

Species	TRU-Araneidae-315	TRU-Araneidae-316	TRU-Araneidae-317	TRU-Araneidae-318
*A.beibeng* TRU-Araneidae-315				
*A.beibeng* TRU-Araneidae-316	0.000			
*A.caesarea* TRU-Araneidae-317	0.161	0.161		
*A.caesarea* TRU-Araneidae-318	0.160	0.160	0.013	
